# Morphogenetic Implications of Peristalsis-Driven Fluid Flow in the Embryonic Lung

**DOI:** 10.1371/journal.pone.0132015

**Published:** 2015-07-06

**Authors:** Kishore K. Bokka, Edwin C. Jesudason, Oswaldo A. Lozoya, Farshid Guilak, David Warburton, Sharon R. Lubkin

**Affiliations:** 1 Department of Mechanical Engineering, North Carolina State University, Raleigh, North Carolina, United States of America; 2 National Health Service (NHS), Edinburgh, Scotland, United Kingdom; 3 Orthopaedic Research Laboratories, Department of Orthopaedic Surgery, Duke University Medical Center, Durham, North Carolina, United States of America; 4 The Saban Research Institute, Childrens Hospital Los Angeles, Los Angeles, California, United States of America; 5 Department of Mathematics, North Carolina State University, Raleigh, North Carolina, United States of America; University of Giessen Lung Center, GERMANY

## Abstract

Epithelial organs are almost universally secretory. The lung secretes mucus of extremely variable consistency. In the early prenatal period, the secretions are of largely unknown composition, consistency, and flow rates. In addition to net outflow from secretion, the embryonic lung exhibits transient reversing flows from peristalsis. Airway peristalsis (AP) begins as soon as the smooth muscle forms, and persists until birth. Since the prenatal lung is liquid-filled, smooth muscle action can transport fluid far from the immediately adjacent tissues. The sensation of internal fluid flows has been shown to have potent morphogenetic effects, as has the transport of morphogens. We hypothesize that these effects play an important role in lung morphogenesis. To test these hypotheses in a quantitative framework, we analyzed the fluid-structure interactions between embryonic tissues and lumen fluid resulting from peristaltic waves that partially occlude the airway. We found that if the airway is closed, fluid transport is minimal; by contrast, if the trachea is open, shear rates can be very high, particularly at the stenosis. We performed a parametric analysis of flow characteristics' dependence on tissue stiffnesses, smooth muscle force, geometry, and fluid viscosity, and found that most of these relationships are governed by simple ratios. We measured the viscosity of prenatal lung fluid with passive bead microrheology. This paper reports the first measurements of the viscosity of embryonic lung lumen fluid. In the range tested, lumen fluid can be considered Newtonian, with a viscosity of 0.016 ± 0.008 Pa-s. We analyzed the interaction between the internal flows and diffusion and conclude that AP has a strong effect on flow sensing away from the tip and on transport of morphogens. These effects may be the intermediate mechanisms for the enhancement of branching seen in occluded embryonic lungs.

## Introduction

The prenatal lung is completely fluid-filled, and epithelial cells lining the lung continuously produce lumen fluid throughout gestation. The fluid is mainly secreted in the distal end and then flows to the upper airway where it is either absorbed or released into the amniotic space. Fluid production increases till mid gestation period and then decreases as the lung reaches full term [1]. The production of lumen fluid helps in maintaining the intraluminal pressure required for normal lung growth. Anything affecting the fluid production or drainage adversely affects lung morphogenesis [2].

Thus, mechanical inputs strongly affect developing lung—but the details matter. What is the load on a tissue? What parts of the tissue are deformed, how much is each location deformed, in what directions is it deformed, and is there a dynamic signature? How might mechanical inputs be sensed by the tissue, in different locations, in different directions, and what might that mean for the adaptive functioning of the organ? How do varying tissue and fluid characteristics affect the mechanical aspects?

**Fig 1:**
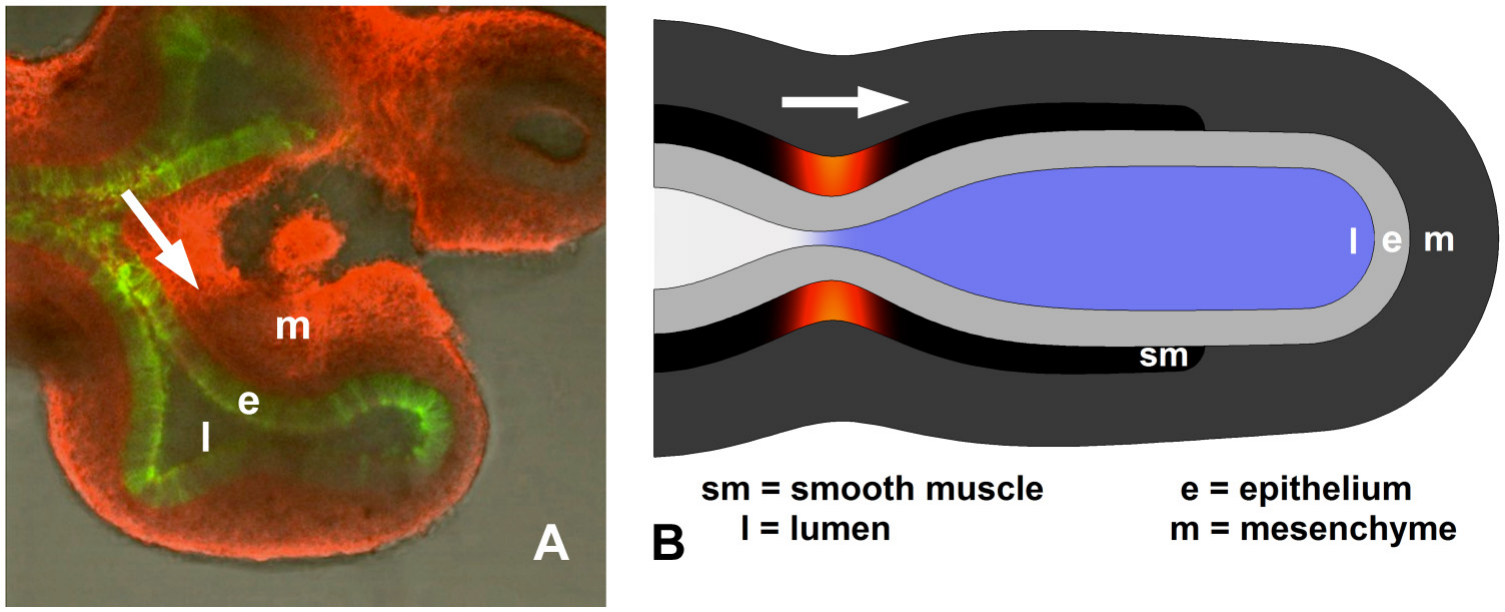
Geometry of embryonic lung and model. A. Explanted E11.5 mouse lung showing lumen (l), epithelium (e, green), and mesenchyme (m, red). Smooth muscle (sm) not visible. B. Embryonic lung idealized as unbranched, axisymmetric tubule, with three uniform tissue layers plus lumen. Smooth muscle undergoes active circumferential contraction wave (red), propagating distally, building lumen pressure ahead of it (blue).

Lung lumen fluid is moved by secretion, airway peristalsis (AP) and, later, fetal breathing movements. Airway peristalsis and tonic contractions begin as soon as airway smooth muscle (SM) forms. AP is initially weak and uncoordinated, but settles into a rhythmic wave moving proximodistally [3] (Fig 1). It has been proposed that AP regulates lung morphogenesis, through tissue stretch [3], the magnitude and direction of which have been quantified [4, 5]. We here propose that the fluid flow generated by AP may itself be significant in prenatal lung development, in several different ways.

Peristalsis in the broader sense has been thoroughly studied in open-ended tubes such as the gut and ureter, where it is an essential method of transport [6–10]. However, there is not, to our knowledge, any previous study of tube peristalsis towards a closed end, as in the lung. Although the smooth muscle action is similar in the gut or ureter (both ends open) and lung (proximal end open, distal end closed), we will show that the flow characteristics are completely different, due to the closed distal boundary and elastic energy storage. The characteristics of the flows in prenatal AP may have several important implications for prenatal lung development.

There is a third distinct case of peristalsis, when both ends of the tubule are closed. In the intact embryonic airway, the larynx is pinched closed, but opens under sufficient lumen pressure. *In vitro*, a trachea excised distal to the larynx will close up in a wound healing response, creating a sealed lumen. The trachea can be maintained in an open configuration by insertion of a stiff tube. These situations of open or closed trachea are mechanically distinct, and will have different characteristics of tissue stretch [4, 5] and flow.

This goal of this study was to analyze the flows of AP in the prenatal lung, using estimates based on standard results from fluid dynamics, measurements of viscosity of prenatal lung lumen fluid, and a computational model of the fluid-structure interactions in a simplified unbranched tubule, with a closed distal end (tip) and either open or closed proximal end (trachea). Our analysis leads us to suggest morphogenetic roles for AP in the two separate arenas of flow sensing and enhancement of transport of solutes such as growth factors. In both cases, we are able to quantify observable measures and suggest expected experimental outcomes.

## Results

### Basic results

#### Zero net fluid flow

In the embryonic lung, the Reynolds number is relatively low (Re = 1), and thus, inertial effects can be neglected. Furthermore, in vitro studies have shown that flows due to AP can be completely reversible, as suspended debris in the lumen has been shown to move in response to AP and return to its original location [3]. In a tube with both ends open, peristalsis is an effective pumping mechanism, common in physiology and industry. However, in AP, the distal ends are closed, so over a complete cycle, the net flow is zero. When the wave ceases, the fluid returns, with a slower reverse flow driven by elastic energy storage of the tissues. Therefore any *net* flow into or out of the lumen must be due to other processes such as secretion (causing outward flow) and growth (causing inward flow). Although AP thus does not serve to remove fluid, it strongly affects diffusive transport, as detailed below.

#### Peristalsis with partial occlusion creates reverse flow (reflux)

**Fig 2:**
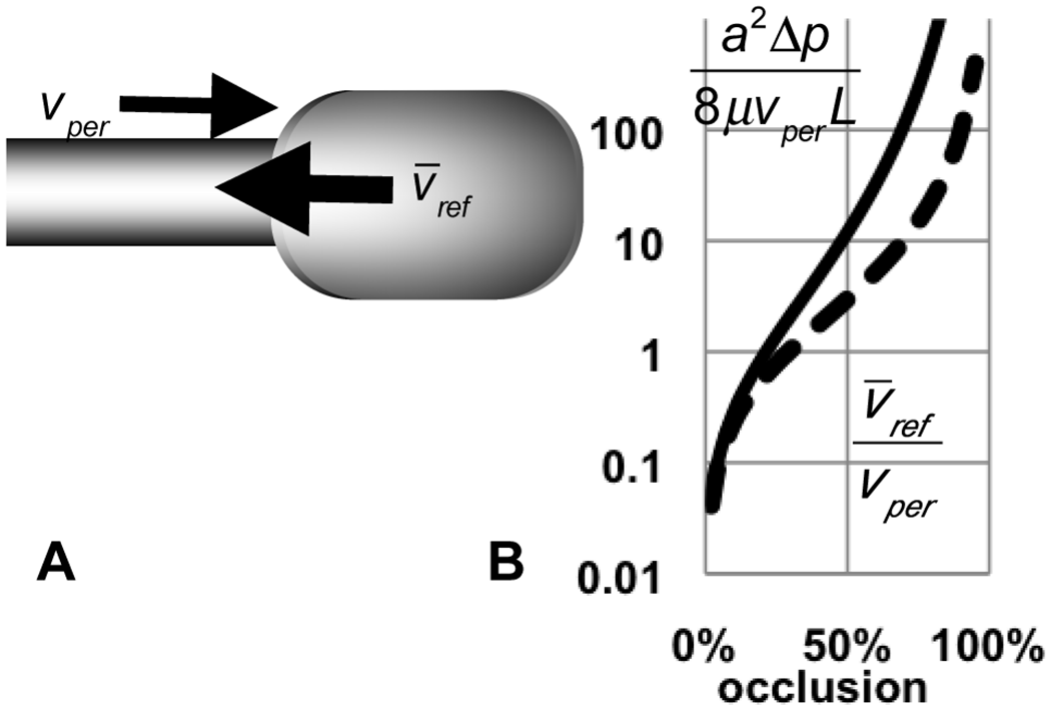
Estimates of reflux velocity and pressure. A. Partial occlusion moving distally pushes fluid proximally (reflux), and creates a pressure gradient across the stenosis. B. At the stenosis, average reflux velocity ${\bar{v}}_{ref}=v_{per}\cdot \left(1-{\left(1-O\right)}^{2}\right)/{\left(1-O\right)}^{2}$ is proportional to velocity of peristaltic wave *v*_*per*_, but increases rapidly with occlusion (dashed curve). Pressure gradient across the stenosis is proportional to fluid viscosity *μ* and strongly depends on occlusion *O*: $\frac{dp}{dx}=\frac{8\mu v_{per}}{a^{2}}\cdot \frac{1-{\left(1-O\right)}^{2}}{{\left(1-O\right)}^{4}}$, where *a* is the relaxed lumen radius (solid curve).

Although the smooth muscle contracts in the same manner, AP moves lumen fluid very differently in the cases of *complete occlusion* (CO) and *partial occlusion* (PO). Most significantly, the direction of flow is different in CO and PO: For CO, fluid moves *with* the peristaltic wave, reversing only when the wave is released. For PO, fluid moves *counter* to the peristaltic wave (Fig 2A), reversing when the wave is released. For CO, the tissue deformations may be very large due to pressure buildup distal to the occlusion [4, 5]. Although tissue deformations can be large in CO, fluid flow rates are small, and there is virtually no fluid shear once occlusion is complete. In contrast, for PO, the tissue deformations are smaller, but the flow past the stenosis is substantial. Because flows from CO are small, in this paper, we focus our analysis of flows only on those driven by partial occlusion.

#### Even modest AP occlusion creates strong reflux

In complete occlusion, fluid moves ahead of the peristaltic wave, in the direction of the wave. In incomplete (partial) occlusion, that flow still occurs at a smaller level, but there is substantial flow in the direction *opposite* to AP in the region of the stenosis. Flow at the stenosis moves opposite to the motion of the AP wave (reflux or counterflow) because the distal tip is closed; if both ends of the tube were open, or in a closed circulation (loop), fluid could flow forwards or backwards.

We estimate the relationship between the velocity of the peristaltic SM wave *v*_*per*_ and the average reflux velocity at the pinch ${\bar{v}}_{ref}$, using the incompressibility of the lumen liquid, and neglecting elastic energy storage in the expanding tip. The velocity ratio is a function of the degree of occlusion *O*: $\frac{{\bar{v}}_{ref}}{v_{per}}=\frac{1-{\left(1-O\right)}^{2}}{{\left(1-O\right)}^{2}}$ (Fig 2). If there is zero AP, there must be zero flow, unless there is some other process such as secretion. Even a small amount of occlusion creates some counterflow; moderate occlusion creates substantial flow. For example, a 50% occlusion of a 50 μm diameter lumen at 70 μm/s [3] would create a counterflow at 200 μm/s with a shear rate of 64/s (assuming a Newtonian fluid). If that occlusion were only 20%, the counterflow would be only 40 μm/s and shear rate 8/s; if occlusion were 80%, the counterflow would be 1600 μm/s and the shear rate an impressive 1300/s (lower if we account for expansion of the tip). At complete occlusion, there is again zero flow at the stenosis.

**Fig 3:**
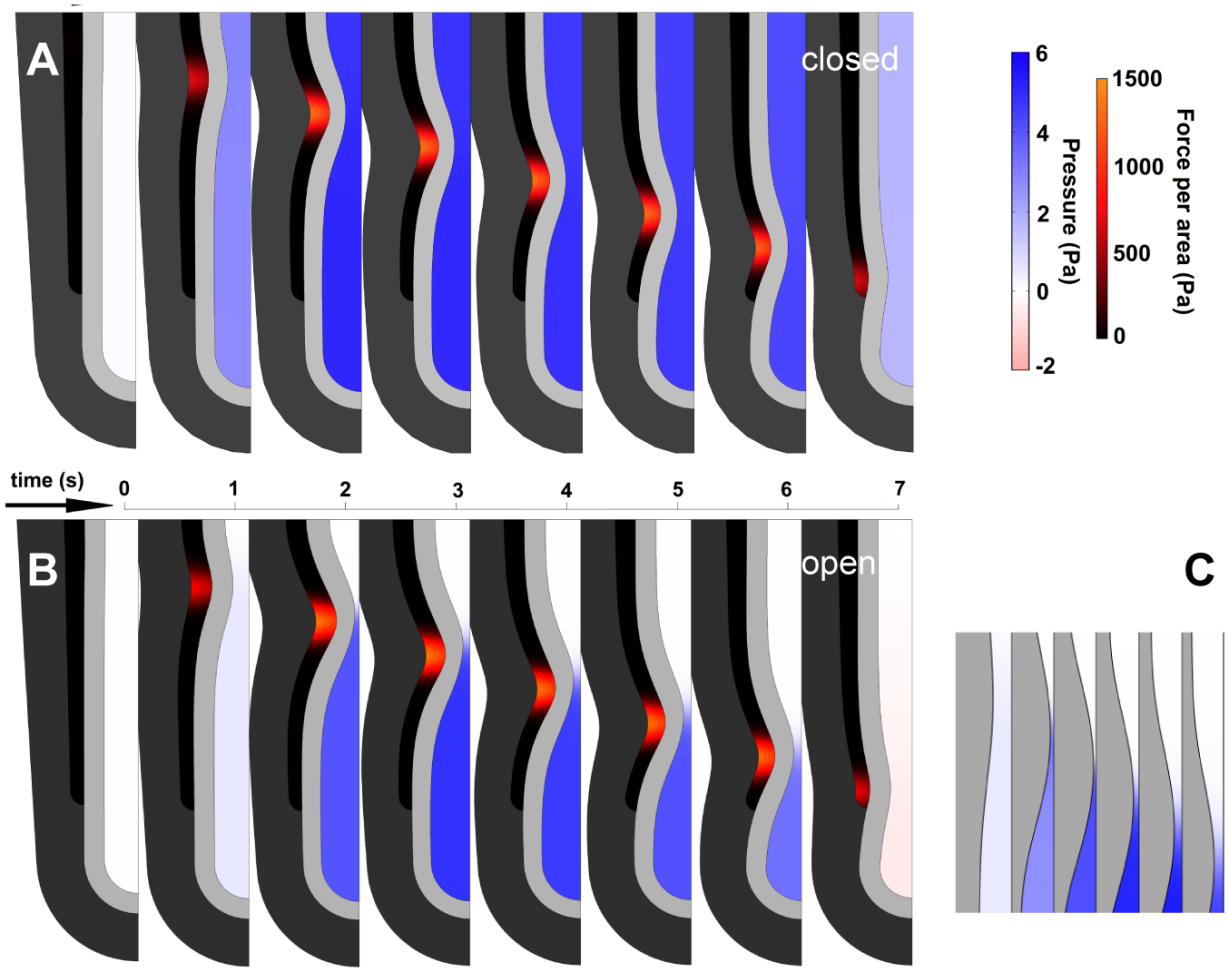
Frames from model simulations of AP with partial occlusion, for open and closed trachea. Each frame shows half the symmetric tubule. A. Closed trachea. Lumen pressure is spatially uniform and increases as soon as AP begins. B. Open trachea. Lumen pressure is negligible until occlusion is almost complete. Pressure is uniform everywhere in the lumen except at stenosis, where flow is fastest. Maximal occlusion shown ~ 90%. C. Detail of open-trachea AP. Maximal occlusion precedes maximal pressure. Pressure distal to pinch forces fluid leakage and reduces occlusion as wave moves distally. Identical parameters (stiffness, viscosity, force input). Frames every 1.0 sec (A, B) and 0.5 sec (C).

Flow is coupled to a pressure gradient. If the trachea is closed, the pressure is spatially uniform (Fig 3A, S1 Video). If the trachea is open, pressure is low proximal to the stenosis and higher distal to it (Figs 1B and 3B, S2 Video). Pressure is uniform distal to the stenosis because there is essentially no flow there. At the stenosis, the flow velocity depends on the occlusion, so, $\bar{v}=\frac{1}{2}v_{mid}=\frac{a^{2}{\left(1-O\right)}^{2}}{8\mu }\frac{dp}{dx}$, where $\bar{v}$ is the local average velocity, *v*_*mid*_ the midline velocity, *a* the initial tube radius, *O* the occlusion, and *μ* the viscosity [6].

#### Closed trachea constrains flow

**Fig 4:**
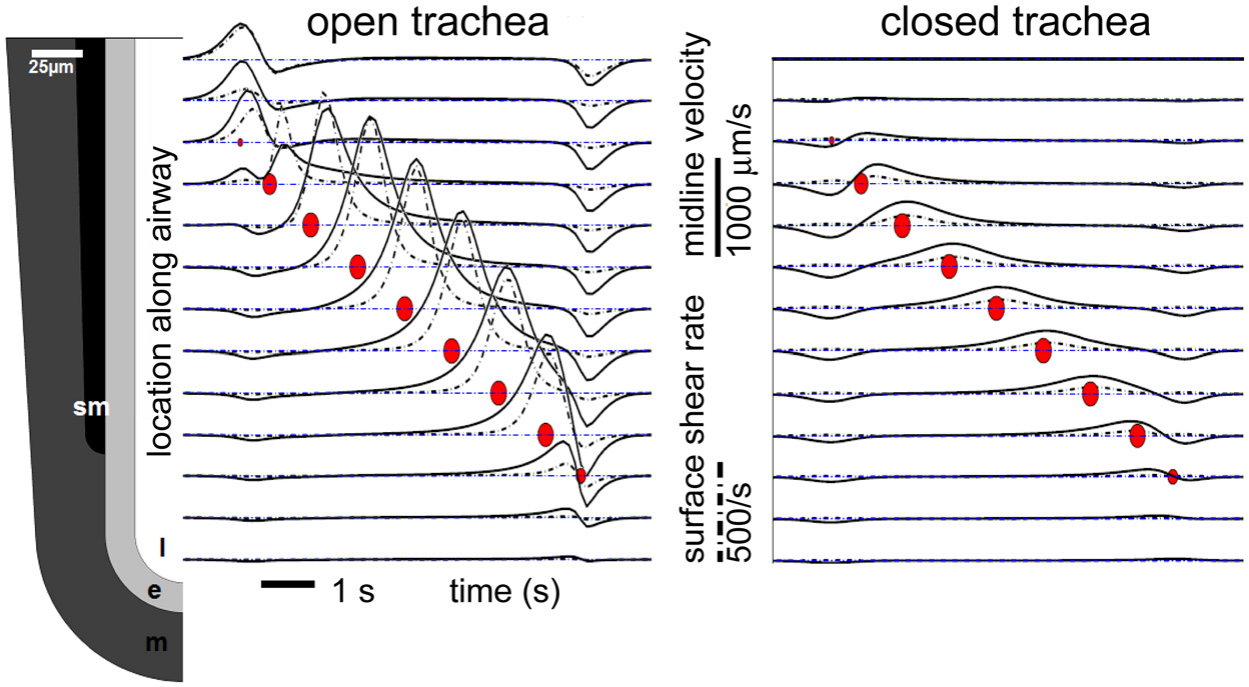
Velocity and shear rate. Lumen fluid velocity at the midline (solid curves) and shear rate at the lumen surface (dashed curves) track each other in time (horiz. axis). Curves correspond to locations on airway at left. Red dots indicate location, relative magnitude, and time of SM force peak. Each curve shows time series of fluid velocity and shear rate. Maximal flow at a position occurs slightly after maximal SM force at that position. Flow is fastest towards trachea, opposite the direction of peristaltic SM wave; refilling flows are slower. Flow distal to SM is negligible. Flow is dramatically reduced in the closed-end airway.

A closed trachea blocks flow out of the lung, so flow inside the lung is substantially reduced. Because a closed trachea does not permit pressure release, lumen pressure is spatially uniform, and occlusion is substantially reduced for the same SM force (Fig 3, S1 Video, Fig 4). If the trachea is open, flow is substantial within the lung, flow is substantial exiting the lung, occlusion is greater, and flow and pressure gradients at the stenosis can be very large (Fig 3, S2 Video, Fig 4).

#### Flow in cross-section

In viscous flow, as in the prenatal lung, the fluid touching the lumen surface does not move relative to the surface (no slip). However, the lumen surface is where we find the maximal fluid shear. Conversely, in a symmetric tubule, the maximal fluid velocity is at the midline of the tubule, where fluid shear is zero. Flow velocities at the midline almost perfectly track shear rates at the epithelial surface (Fig 4).

### Viscosity

#### Dependence of flow on measurable parameters

We performed a parametric study of our computational model to determine the relationship between model inputs and outputs. Some flow features are independent of the viscosity of the lumen fluid. Flow is driven by pressure buildup due to occlusion. Maximal occlusion *O* is linear in the ratio *FT/E*, where *F* is SM force density, *T* is epithelial thickness, and *E* is tissue stiffness. For a closed trachea, our simulations gave *O* = 0.46*FT*/*E* (R^2^>0.99); for open trachea, *O* = 0.67*FT*/*E* (R^2^>0.98), independent of viscosity. Pressure buildup from AP depends on fluid viscosity only if the trachea is open. For closed trachea, maximal pressure depends only on smooth muscle force density *F* and epithelial thickness *T*: *p*_max_ = 0.087*FT* (R^2^>0.99); for open trachea, maximum pressure is roughly linear in viscosity: *p*_max_∝(*μ*/*μ*_*w*_)^0.8^ (R^2^>0.96), consistent with our estimates illustrated in Fig 2B.

#### Rheometry

**Fig 5:**
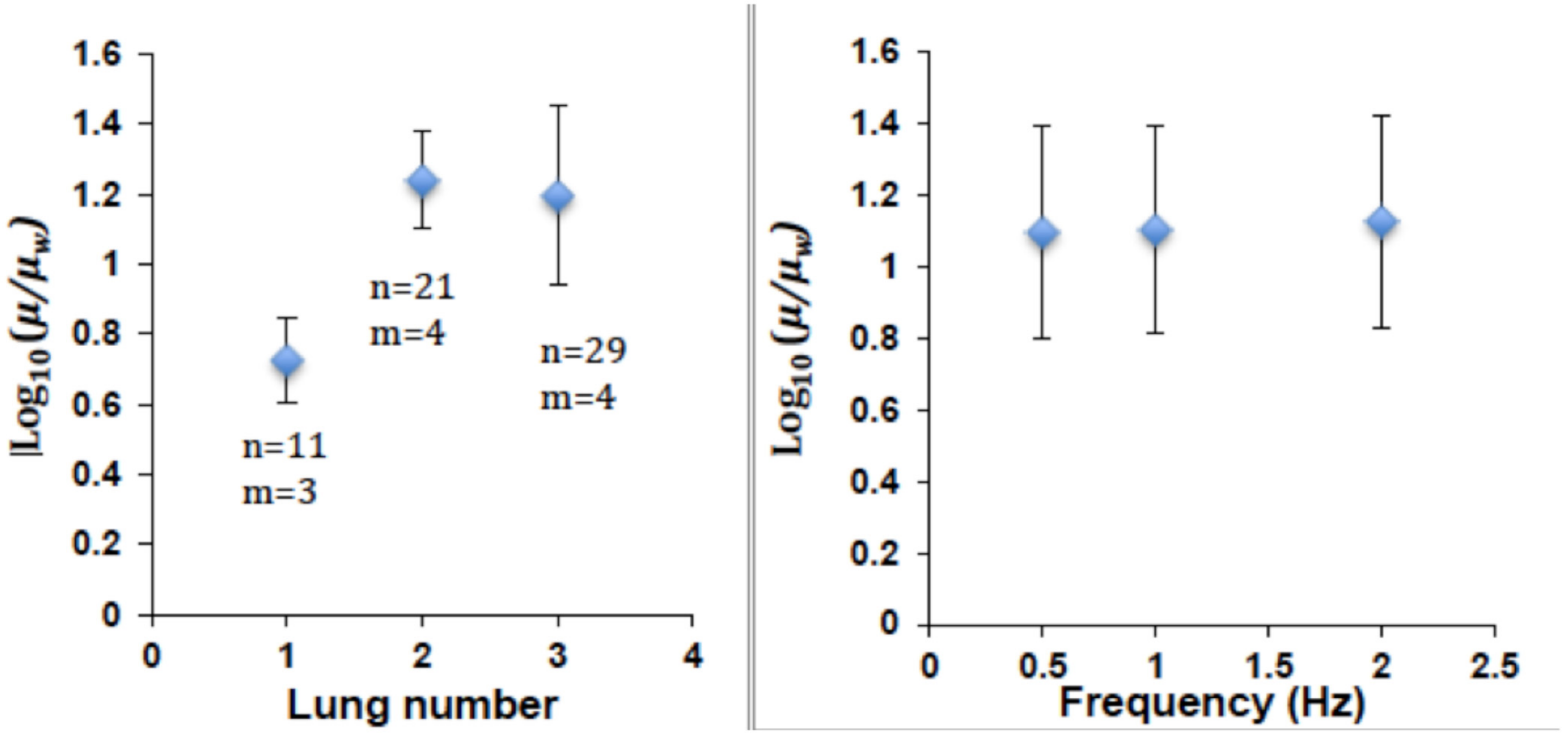
Viscometry of lumen fluid from prenatal mouse lungs. Ratio of lung fluid viscosity to viscosity of water is 1.1 ± 0.3. Variability between individuals (*N* = 3) is greater than variability between beads (*n* = 11–29), locations in lung (*m* = 3–4), or frequency (0.5–2 Hz).

Using particle-tracking microrheometry [11] with injected 500 nm beads, we determined the properties of the lumen fluid in 3 prenatal mice from different litters. The slopes of the log-log plots of MSD against time interval (in the range 0.2–30 s^-1^) did not measurably differ from 1 (*R* ^2^ > 0.95). Thus, in the prenatal mouse lungs measured, the lumen fluid was Newtonian up to 30 s^-1^. We found that the viscosity of the lung fluid is an order of magnitude higher than that of water (log_10_(*μ*/*μ*_*w*_) = 1.1±0.3). Specifically, water has a viscosity of 0.001 Pa-s and we measured embryonic mouse lung lumen viscosity at 0.016 ± 0.008 Pa-s. Variability between individuals was significantly greater than variability within an individual or between frequencies (Fig 5).

### Mixing

#### Bulk mixing

**Fig 6:**
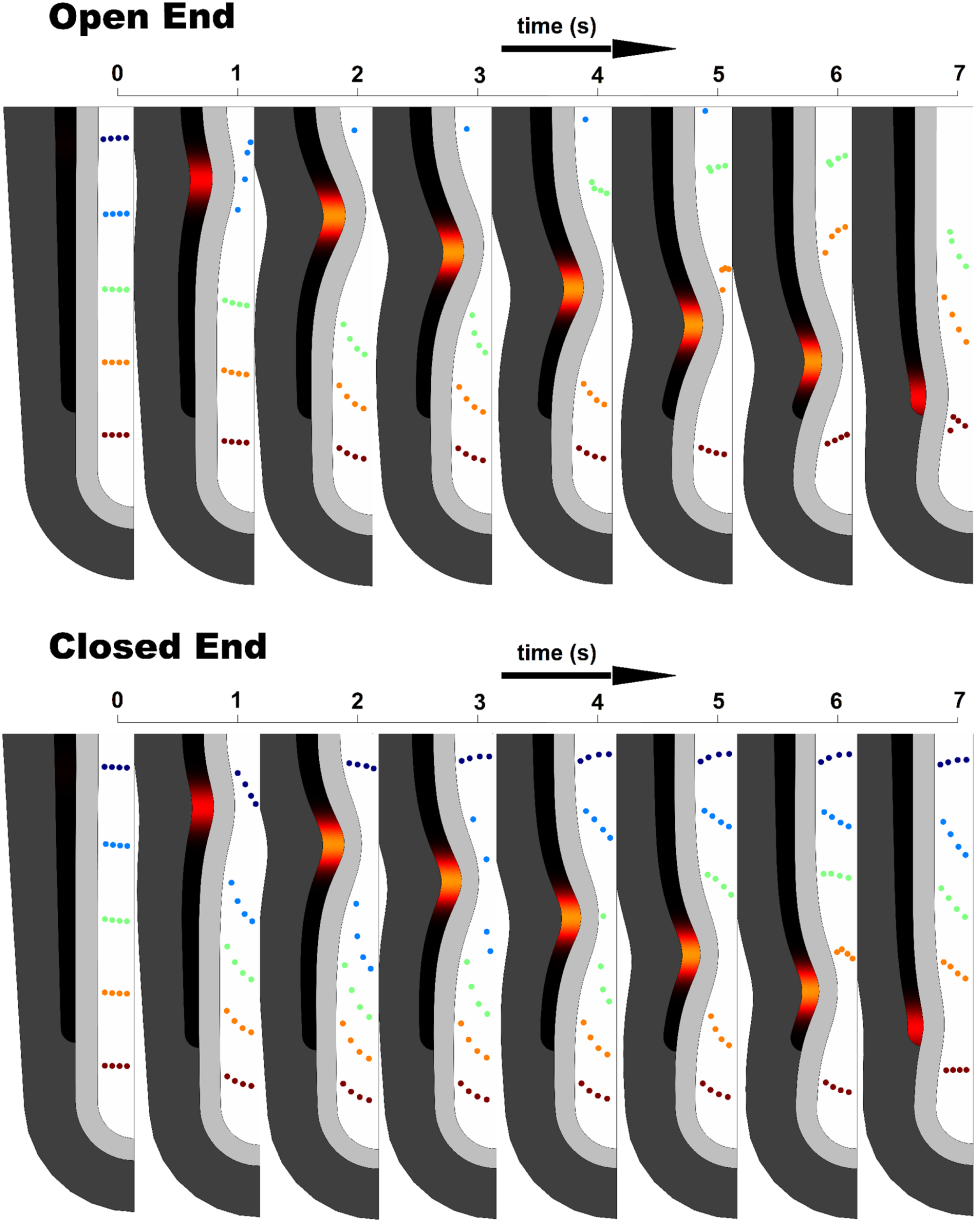
Peristaltic wave dramatically stretches fluid layers adjacent to the occlusion, while modestly affecting distal fluid. If the trachea is open, mixing is much more dramatic than if the trachea is closed. Even for the closed trachea, fluid markers do not return precisely to their original locations despite the low Reynolds number. The spatiotemporal asymmetry of the waveform results in mixing.

Although embryonic AP is in the Stokes flow regime Re≪1, implying reversibility of the flow [12], the waveform is asymmetric and does not generally reverse. Because of the non-reversing waveform, the lumen fluid undergoes a certain amount of mixing (Fig 6, S3 and S4 Videos). If the trachea is closed, the lumen fluid remains in the lumen, but undergoes a small amount of mixing. If the trachea is open, in AP as in breathing, a large amount of fluid can exit the lung and mix with external fluid before refilling the lung. Regardless of the state of the trachea, there is essentially no mixing at the distal airway tips, because fluid shear is small distal to the stenosis.

#### Diffusion

At small length scales, as in the embryo, diffusion is the dominant mode of transport. Diffusion coefficients vary over many orders of magnitude depending on the size of the molecule and the viscosity of the fluid in the lumen. The Stokes-Einstein relation *D* = *kT*/6*πμr* gives the diffusion coefficient *D* as a function of the molecular radius *r* and the fluid viscosity μ. The possible range of diffusion coefficients relevant to AP is large (Table 1) because of the potential range of viscosities and molecular sizes. The time to diffuse a distance *x* is proportional to *x* ^*2*^ */D*, or, using the Stokes-Einstein relation above, the diffusion time is proportional to *x* ^*2*^ *μr*. Thus in the embryonic lung, where the lumen fluid viscosity is somewhat greater than water but smaller than mucus, the time scale of molecular diffusion at a length scale of 100 μm is roughly 1 minute for CO_2_ and 30 minutes for a 100 kDa globular protein. In contrast, CO_2_ in water diffuses 100 μm in 5 s and a 100 kDa protein diffuses 100 μm in neonatal mucus in 10 hr. Because diffusion time scales as the *square* of the distance, diffusing 1 mm in the embryonic lung lumen will take 2 hrs for CO_2_ and 2 days for a 100 kDa protein. These time scales are clearly limiting in both metabolism and development.

**Table 1:** Diffusion coefficients (μm^2^/s) of various molecules in various fluids.

	water	embr lung lumen fluid (meas)	mucus, neonate [13]	mucus, adult [14]
CO_2_	2E+03	1E+02	5E+00	7E-04
glucose	6E+02	4E+01	2E+00	2E-04
100 kDa glob protein	1E+02	6E+00	3E-01	3E-05

Diffusion coefficients in μm^2^/s. Viscosities (Pa-s) of water 0.001, mouse embryonic lung lumen fluid (this paper) 0.016, neonatal mucus 0.4, adult mucus 3000.

#### Taylor dispersion: interaction of mixing and diffusion

As soon as the lung reaches a size for which diffusion becomes too slow to satisfy its transport needs, the smooth muscle forms and AP begins. Fluid motion dramatically enhances transport. Solutes and suspended particles advect a distance *x*, in a flow of average speed $\bar{v}$, on a time scale $x/\bar{v}$. A flow at the modest $\bar{v}$ of 50 μm/s travels 100 μm in 2 s, and 1 mm in 20 s. We have shown that even a small occlusion *O* from AP at speed *v*_*per*_ can create a substantial reverse flow $\bar{v}=v_{per}\left(1-{\left(1-O\right)}^{2}\right)/{\left(1-O\right)}^{2}$ which exceeds *v*_*per*_ for any occlusion > 29% (Fig 2).

**Fig 7:**
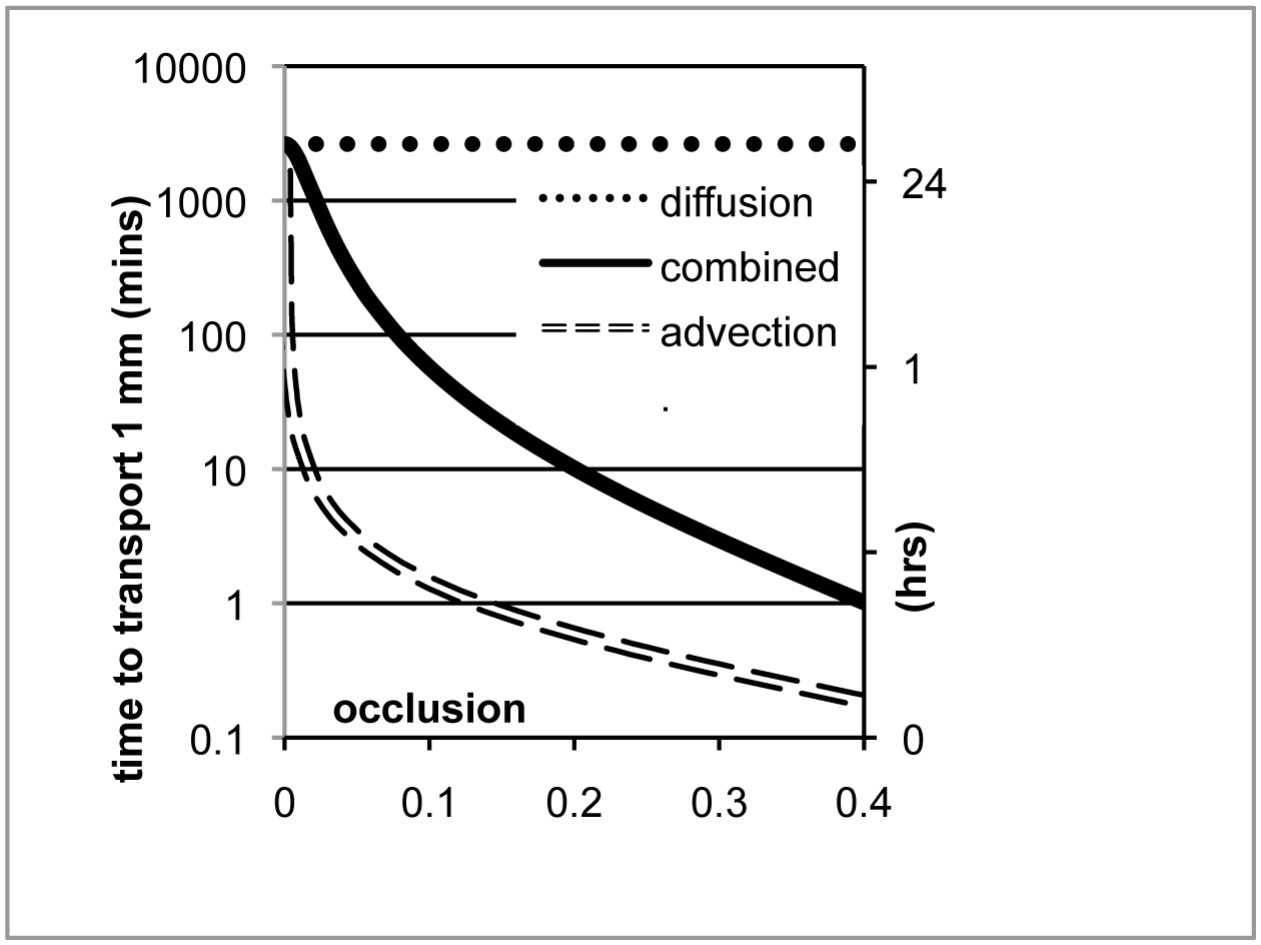
Time scales of transport in the embryonic lung. In the absence of flow, solutes can only diffuse (dotted curve). In the absence of diffusion, solutes and particles advect with the flow (dashed curve). Advection-diffusion (solid curve) transports solutes rapidly relative to diffusion alone, and a small occlusion from weak airway peristalsis can yield a dramatic reduction in transport time. 100 kDa globular protein in lumen fluid of measured viscosity 0.016 Pa-s.

When AP begins, it is weak, and occlusions are small. The reflux velocity will be correspondingly smaller, and advection and diffusion interact in the intermediate range of Taylor dispersion [15, 16]. In this regime, solutes diffuse with an effective diffusion coefficient $k=a^{2}{\bar{v}}^{2}/48D$, where *a* is the lumen radius, bounded by the molecular diffusion coefficient *D*, giving $k∼D\left(1+\frac{1}{48}{\left(a\bar{v}/D\right)}^{2}\right)$ (Fig 7). For example, for a 100 kDa globular protein in the embryonic lung lumen with viscosity 0.016 Pa-s and tubule inner radius 25 μm, the time scale to diffuse 100 μm is 30 minutes, but 1 mm takes 2 days. With a peristaltic wave at 50 μm/s and just 10% occlusion, yielding $\bar{v}=12$ μm/s, that 100 kDa protein is transported 1 mm in an hour instead of 2 days, because of the interaction between advection and diffusion.

Thus AP can substantially enhance diffusive transport even for a small occlusion. The enhancement is greater, the larger the molecule and the more viscous the fluid. The effect is small for diffusion in water, but for diffusion in mucus, the effect can be very large.

## Discussion

### Airway peristalsis coupled with flow sensing may serve to optimize lung allometry

Branching in the pseudoglandular period proceeds on a consistent length scale. It is only in later developmental stages that differential growth rates and oriented cell divisions [17] lead to the correct scaling of the branch generations, optimized for efficiency of transport [18]. It is well established that the vascular system uses shear sensing to detect flow rates and regulates wall thickness in response. It may be that one of the primary morphogenetic functions of AP is to generate internal flows to stimulate widening of bronchi in response to shear stresses, in order to optimize branch allometry. We hypothesize that AP provides a fluid mechanical stimulus to the developing airway, allowing adjacent tissues to sense flows, and thus to respond with differential growth rates and/or orientationally biased cell divisions, in order to minimize surface shear.

### Flow detection by surrogate measures

A cell cannot directly detect bulk fluid velocity, but it can infer bulk velocity from the surrogate measure of shear rate at the lumen surface. In a straight tube, the maximal fluid velocity is on the midline, and there is zero flow at the lumen surface. Conversely, fluid shear is zero on the midline, and maximal on the lumen surface. At a given location along the airway, for our assumption of a Newtonian fluid, surface shear rate is nearly proportional to midline velocity (Fig 4), except for small lags from occlusion transients. Thus, if it exists, epithelial flow sensing at the lumen surface would be a surrogate mechanism for sensing flow away from the tissue surface, i.e. at the midline. Conversely, for the experimenter, tracking the velocity of debris at the midline provides a surrogate measure for shear rates at the epithelial surface.

### Flow sensing in tips?

In all cases, shear rates from AP are negligible in the tip region, only becoming significant in the part of the airway experiencing SM contraction (Fig 4). Primary cilia (PC) appear in proximal airways by E12.5, before motile cilia, and are gradually replaced by motile cilia through at least E15.5; they reappear later in response to injury [19]. PC regulate branching morphogenesis in mammary glands [20]. Because AP contributes negligible shear in the region distal to the SM, we predict that flow-sensing mechanisms such as primary cilia will not be found distal to SM in the embryonic lung.

We also predict that any flow-sensing PC would be shorter proximally, where the SM is most strongly contractile and flows are fastest, and longer distally, where SM, SM contraction, and flows are less developed. If there were flow in the distal tip, it could not be due to AP, but must be due to secretion. If flow-sensing primary cilia are found distal to the SM (PC are found in mammary terminal end buds [20]), we can conclude that they are there in order to detect secretion distal to their location, not to detect flows generated by AP.

Thus, if PC are found along the entire intraluminal surface of a growing bud, they may perform two separate functions in the developing airway, depending on their position: either probing AP-driven flow in the SM-wrapped epithelium; or detecting secretion-driven flow at the distal tip. In particular, because shear from AP is greater where SM is more developed (i.e. proximally), intraluminal PC should also be progressively shorter if SM-associated epithelial cells are to perform equal physiological functions, since signaling at PC depends on the scale of bending and stretching of its plasma membrane that are proportional to fluid shear. By the same principle, distal tip epithelial cells should have PC with a mechanotransduction machinery fine-tuned to secretion instead of AP-driven flow. This suggests that signaling networks in distal tip epithelia would be functionally distinct from those in SM-associated epithelia and, consequently, that epithelial cells from either of those two regions should be phenotypically distinct.

We speculate that PC serve to sense flow in developing ducts, and in turn to regulate morphogenesis based on sensed flows (some examples are reviewed in [21]). Because of spatiotemporal asymmetry in lumen flows, PC could potentially discern positional information by flow sensing alone, and further, could distinguish between flows generated by secretion (unidirectional) and flows generated by AP (bidirectional, with a characteristic waveform).

### Positional information

If an epithelial cell were able to detect stretch at its basal or apical end, and flow at its apical surface, it could, in principle, infer information from far away in the lung, using only local information. It could, in effect, remotely detect, from hundreds of microns away, whether the trachea is closed or open. For example, an airway epithelial cell that sensed stretch could infer its position from geometric cues (discussed in [5]), and if it also sensed no fluid shear accompanying the stretch, despite proximity to contracting SM, it could infer that the airway is proximally blocked. Conversely, if an epithelial cell detects fluid shear without tissue strain (from adjacent SM contraction), it could infer, from mechanical cues alone, distant distal secretion.

### Airway peristalsis and bulk transport

Smooth muscle forms in the lung at the same time as the lung becomes too large for diffusion to remain an efficient means of transport. At the same time, the cardiovascular system is developing, and is also moving fluid by peristalsis. Peristalsis represents an ancient and widespread mechanism for bulk transport. We have shown that even a small, weak peristaltic wave in the embryonic airway can dramatically increase the efficiency of transport, especially for large molecules, and especially in viscous fluids, such as may develop in late gestation. Thus the known effects of AP in stimulating and regulating morphogenesis may be based not just in mechanisms involving tissue stretch, but also in mechanisms involving transport of morphogens. Enhanced transport especially of large molecules, such as growth factors, in the lumen would have the effect not just of enhancing transport *to* proximal tissues, but also of enhancing transport *from* distal tissues.

### Viscometry

This paper reports the first measurements of the viscosity of embryonic lung lumen fluid. We found that in the range tested (0.2–30 s^-1^), lumen fluid could be considered Newtonian. Our model and analysis were built on an assumption of the unknown properties of the lumen fluid being Newtonian. Shear rates in AP can be high (100-1000/s), as we find both from our estimates without elastic energy storage and also from our simulations with elastic energy stored in the tissues. The highest shear rates are at the stenosis; the closer the occlusion is to complete, the higher will be the shear rates at the stenosis. If the lumen contains mucus, its non-Newtonian properties will be significant in this flow regime. If further testing reveals non-Newtonian behavior at higher shear rates or in other model organisms, we might then conclude that one of the many functions of prenatal AP is the same function as coughing and sneezing in the postnatal lung: to exploit the shear-thinning properties of mucus, to clear it from the lung.

### Model validity

Although errors in computing advection are well known, the fundamental reason that fluid tracers do not return to their original positions (Fig 6, S3 and S4 Videos) is not computational error, but asymmetry of the waveform of the moving boundary.

### Control of morphogenesis by multiple interacting factors

Mechanics interacts with cell signaling in numerous complex subsystems (reviewed in [22–24]). AP does many things all at once—stretching tubule cells apicobasally [4, 5], flattening tip cells [4, 5], transporting solutes, shearing surface receptors—so the potential morphogenetic mechanisms are confounded. Thus, experiments which only inhibit or enhance AP cannot tease apart the multiple mechanical effects that AP has on a developing tissue. However, with the framework provided by this study and others [4, 5], individual components of mechanical stimulation such as tissue stretch and fluid sensing can be isolated, for example by altering lumen fluid viscosity. Other factors not analyzed in this study will also interact with AP and confound results. For example, it would be useful to quantify the pressure and secretion rate in the embryonic lung, as has been done in the later lung [1, 2, 25]. In another paper [26], we estimate the secretion rate in the embryonic lung to yield an exit velocity an order of magnitude lower than that from AP.

## Conclusions

We know that mechanical inputs strongly affect developing organs—but the mechanical details matter, and are studied far more rarely than the molecular details: what is the load on a developing tissue, what parts of the tissue are deformed, how much is each location deformed, in what directions is it deformed, and is there a dynamic signature? Might mechanical inputs be sensed by the tissue, in different locations, in different directions, and what might that mean for the adaptive functioning of the organ? Do varying tissue and fluid characteristics affect the mechanical aspects? In the developing lung, for example, it has long been appreciated that fetal lung liquid plays a key role in morphogenesis and development: draining the liquid leads to tissue hypoplasia, whereas blocking the exit leads to dysplastic overgrowth of the tissue. There have even been clinical trials in humans to evaluate whether the latter approach might be useful to rescue hypoplastic lungs in utero, some with positive and some with mixed results. This paper thus considers the development of the lung from the point of view of fluid mechanics and provides some important insights from first principles in this field. While fluid mechanics is not on the usual syllabus for pediatricians or pediatric surgeons, clearly it has been co-opted by evolution to help form the branched structure and gas diffusion surface of the developing lung.

## Methods

### Computational model

The embryonic lung was modeled as a single unbranched radially symmetric tubule with three tissue layers and a lumen (Fig 1B).

The tissues were modeled as uniform, isotropic, Hookean, undergoing finite strain. The lumen fluid was modeled as uniform, Newtonian, and creeping. The trachea was modeled as either open or closed. To our knowledge, there are no published studies of the mechanical properties of embryonic lung tissues or lumen fluid; we used the estimated parameter values in Table 2. Where a range of values is listed, we tested that range within the limits imposed by numerical convergence. The peristaltic wave was modeled as a distributed contractile body force in the smooth muscle layer, with gradual onset and release, moving distally with constant velocity.

**Table 2:** Parameters and variables used in estimates and computational model.

quantity	symbol	units	range	references
tissue stiffness	*E*	Pa	20–400	[27, 28] ^1^
lumen viscosity	*μ*	Pa-s	10^−3^–10^−1^	[13]^2^
contraction	*F*	pN/μm^3^	0.75–36	[29]^3^
lumen diameter		μm	15–50	
epithelium thickness	*T*	μm	15	
wavespeed	*v*_*per*_	μm/s	67	[3]
occlusion	*O*	-	40–50%	[3]^4^
lumen pressure	*p*	Pa	100–400	[3, 30] ^5^ ^,^ ^6^[31]^7^

^1^ No studies report stiffness of embryonic lung tissue. Range is an estimate. Lower bound: 20 Pa for amphibian embryos; upper bound 400 Pa for ASM cells in vitro.

^2^ We assume that the viscosity of airway lumen fluid in the embryo is lower than that of neonatal airway mucus but higher than that of blood.

^3^ Fetal pig airway SM 1–20 kPa, highest in trachea, lowest in bronchioles. We assume this as an upper bound, and that embryonic SM will likely be weaker by 1–2 orders of magnitude. We assume a SM thickness of 15 microns.

^4^ Fetal pig, pseudoglandular stage

^5^ Fetal mouse (lowest value).

^6^ Rabbit fetus, static pressure.

^7^ Fetal sheep, static pressure.

The dynamic model was implemented in a finite element package, COMSOL, with its Fluid-Structure module, bidirectional coupling, large-strain formulation, ALE moving mesh, and particle tracing. Poisson's ratio in the tissue was approximated by 0.45. Convergence generally required occlusion to be < 95% and maximal lumen pressure < 15 Pa. The FEM model was verified against analytical results for a hollow sphere and an open tube.

### Rheometry

Mice were maintained as a colony in a facility that is accredited by the Association for Assessment and Accreditation of Laboratory Animal Care. All animal procedures were approved by the North Carolina State University institutional animal care and use committee, #12-044-B. Mouse lungs were extracted from discarded C57BL/6J embryos decapitated for a separate study, and kept in PBS. Viscosity of lumen fluid was measured by passive bead microrheometry [32, 33]. Lung extraction, microinjection and particle tracking were done on the same day. 500 nm beads were delivered in the intraluminal space of terminal buds in several areas, by microinjection at 0.2–1 μl intervals. Due to opacity of older tissue, beads were not injected into upper airways. (Beads: Life Technologies FluoSpheres, Carboxylate-Modified, red fluorescent (580/605), quenched in 50 mM Tris pH 7.4 for 15 min at ambient temperature, collected by ultracentrifugation at 15,000×g and 4°C, and resuspended in molecular biology-grade water to about 0.5–1 μg/ml for delivery. Microinjection setup: 100-μl Drummond Positive Displacement Microdispenser, adapted onto an inverted phase contrast/epifluorescence Nikon Diaphot 300 microscope equipped with a Nikon 20X/0.4NA DL Ph2 objective, using borosilicate glass pipetting capillaries previously hand-drawn under heat to microneedle tip barrel diameters between 15–50 μm confirmed by light microscopy.) Bead random walks were recorded in 2D and analyzed with Video Spot Tracker software and 3DFM Matlab module (Center for Computer Integrated Systems for Microscopy and Manipulation (CISMM), UNC Chapel Hill). Because SM activity caused fluid flow in lungs, drift terms were subtracted from particle positions, to yield traces of Brownian motion.

## Supporting Information

S1 File Velocimetry data. Raw data from particle-tracking velocimetry.
(RAR)

S1 Video Peristalsis with closed end. When trachea is closed, pressure in lumen is uniform as peristaltic wave progresses.
(AVI)

S2 Video Peristalsis with open end. When trachea is open, pressure builds distal to stenosis, but remains low proximal to it, which permits greater occlusion than with closed trachea.
(AVI)

S3 Video Particle tracing with closed end. Closed trachea restricts flow of lumen fluid, resulting in minimal mixing of contents. Tracer particles return almost to original positions.
(AVI)

S4 Video Particle tracing with open end. Peristalsis with open trachea creates high shear, resulting in substantial fluid mixing as packets of fluid from near the midline make large excursions, in some cases exiting the lung before lung refills.
(AVI)
